# Distinct properties of semiquinone species detected at the ubiquinol oxidation Q_o_ site of cytochrome *bc*_1_ and their mechanistic implications

**DOI:** 10.1098/rsif.2016.0133

**Published:** 2016-05

**Authors:** Rafał Pietras, Marcin Sarewicz, Artur Osyczka

**Affiliations:** Department of Molecular Biophysics, Faculty of Biochemistry, Biophysics and Biotechnology, Jagiellonian University, Kraków, Poland

**Keywords:** cytochrome *bc*_1_, complex III, ubiquinol oxidation, semiquinone, Q cycle, mitochondria

## Abstract

The two-electron ubiquinol oxidation or ubiquinone reduction typically involves semiquinone (SQ) intermediates. Natural engineering of ubiquinone binding sites of bioenergetic enzymes secures that SQ is sufficiently stabilized, so that it does not leave the site to membranous environment before full oxidation/reduction is completed. The ubiquinol oxidation Q_o_ site of cytochrome *bc*_1_ (mitochondrial complex III, cytochrome *b*_6_*f* in plants) has been considered an exception with catalytic reactions assumed to involve highly unstable SQ or not to involve any SQ intermediate. This view seemed consistent with long-standing difficulty in detecting any reaction intermediates at the Q_o_ site. New perspective on this issue is now offered by recent, independent reports on detection of SQ in this site. Each of the described SQs seems to have different spectroscopic properties leaving space for various interpretations and mechanistic considerations. Here, we comparatively reflect on those properties and their consequences on the SQ stabilization, the involvement of SQ in catalytic reactions, including proton transfers, and the reactivity of SQ with oxygen associated with superoxide generation activity of the Q_o_ site.

## 1. Introduction

Cytochrome *bc*_1_ is one of the key enzymes of respiratory and photosynthetic electron transport chains. The enzyme couples electron transfer between ubiquinone/ubiquinol and cytochrome *c* with proton translocation^1^ across the membrane. Typically, the transfer of electrons from ubiquinol to cytochrome *c* contributes to generation of protonmotive force used for adenosine triphosphate synthesis (for recent reviews, see [1,2]). However, in some cases, the direction of electron flow through cytochrome *bc*_1_ can be reversed, leading to oxidation of cytochrome *c* and reduction of ubiquinone [3,4].

**Figure 1.**
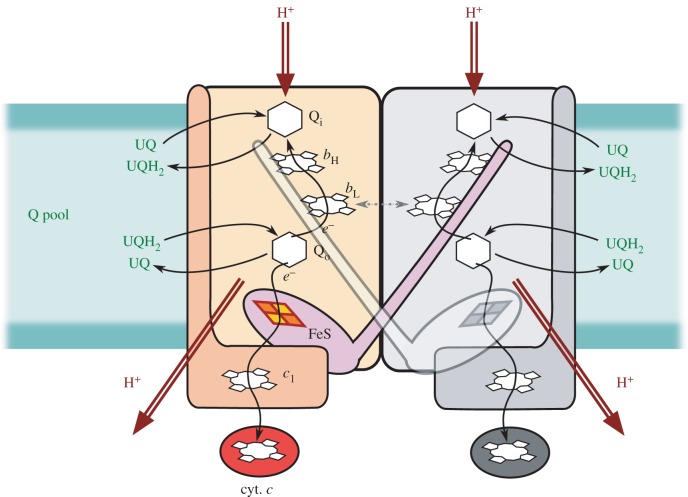
Diagram of homodimeric cytochrome *bc*_1_ structure describing the general mechanism of enzymatic turnover. The ubiquinone binding sites Q_i_, Q_o_ together with haems *b*_L_ and *b*_H_ (*b*-chain) are embedded in cytochrome *b* subunit (light orange rectangle). The Rieske protein (light magenta) harbouring 2Fe–2S (FeS) iron–sulfur cluster and cytochrome *c*_1_ subunit (dark orange) with haem *c*_1_ transfer the electrons from Q_o_ site to cytochrome *c* (red). The proton uptake and release is indicated by red arrows. The intermonomer electron transfer at the level of two haems *b*_L_ [5,6] is indicated by dashed arrow. For clarity, the second monomer is shown in grey. Ubiquinone (UQ) and ubiquinol (UQH_2_) constitute the Q pool.

The translocation of protons across the membrane involves two types of ubiquinone-binding sites facing opposite sides of the membrane: one site oxidizes ubiquinol, whereas the other reduces ubiquinone (figure 1). The joint action of these sites defines the basis of catalytic Q cycle. To secure energetic efficiency of this cycle, the ubiquinol oxidation site (the Q_o_ site) directs electrons into two separate cofactor chains. One electron is used to reduce cytochrome *c*_1_ via electron transfer through the Rieske cluster (FeS) and haem *c*_1_ in one cofactor chain (the c-chain), whereas the other electron is transferred across the membrane to the Q_i_ site via two haems *b* (haem *b*_L_ and *b*_H_ of the b-chain).

The idea that oxidation of ubiquinol in complex III directs electrons into two separate chains, one involving cytochrome *b* and the other cytochrome *c*, was introduced by Wikström & Berden in 1972 [7]. It emerged from a number of earlier observations documenting the intriguing effect of oxidant-induced haem *b* reduction in the presence of antimycin (inhibitor of the Q_i_ site) (see [7] and references therein). This idea was preceded by a tentative scheme published in 1967 by Baum *et al*. [8], who also proposed two separate electron acceptors of ubiquinol, but in that work the connection between the two chains of cofactors was not yet understood. In 1975, Peter Mitchell adopted the idea of Wikström & Berden [7] and introduced the cyclic arrangement of electron transfer through the protonmotive Q cycle featuring two quinone binding sites (as we now know Q_o_ and Q_i_ sites), each standing at a divide of two cofactor chains [9,10]. In 1983, the Q cycle was modified by Crofts *et al*. [11], who realized that electrons for ubiquinone reduction at the Q_i_ site both come from the same cofactor chain, leaving Q_o_ as the only site separating the route for two electrons upon catalysis.

The reaction at the Q_o_ site, often referred to as a bifurcation, is unusual in biology. Its mechanism is still a matter of intense debate. The lack of crystal structures containing native ubiquinone molecule bound in the Q_o_ site [12] and a long-standing difficulty in spectroscopic identification of the intermediate states of the Q_o_ site catalysis have left a high degree of freedom for mechanistic considerations [13–21].

Typically, because of the two-electron nature of ubiquinol oxidation or ubiquinone reduction, a semiquinone (SQ) species is expected to be formed as an intermediate of the reaction [22,23]. Indeed, such intermediates were detected by electron paramagnetic resonance (EPR) spectroscopy in several quinone binding sites, including the Q_i_ site of cytochrome *bc*_1_ [24–26], the Q_B_ site of photosynthetic reaction centre, and quinone sites of mitochondrial complex I and II (reviewed in [27–29]). All those sites are connected to a single chain of cofactors and, consequently, the two-electron oxidation/reduction of QH_2_/Q must proceed step-wise involving a relatively stable and manageable for experimental trapping SQ intermediate. However, the architecture of the Q_o_ site creates distinctly different conditions for ubiquinol oxidation: the substrate binds in between the two chains of cofactors and thus can experience simultaneous presence of two redox centres (FeS cluster and haem *b*_L_) ready to engage in electron transfers. In this case, the two-electron reaction does not need to proceed through the relatively long-lived SQ intermediate. With this simultaneous access to the two electron paths, a detection of SQ intermediate has proven difficult.

One of the early attempts of detection of a semiquinone radical within the Q_o_ site (SQ_o_) by equilibrium redox titration failed to detect a radical signal in CW EPR spectra of redox-poised bacterial chromatophores [30]. In mitochondrial system, the first report of detection of SQ_o_ [31] was questioned in later work [32] which led to a commonly accepted view that detection of this species, if it exists, falls beyond the limits of EPR sensitivity. This has been considered as confirmatory of Mitchell's original idea that the stability constant of SQ_o_ (*K*_s_) must be less than unity. However, recently three groups reported a detection of a SQ at the Q_o_ site [33–36]. Intriguingly, each of the described SQs seems to have different spectroscopic properties. Additionally, the conditions in which they were trapped and subsequently detected by EPR were different. Here, we summarize those reports focusing on comparison of SQ species with respect to their interactions with paramagnetic cofactors of cytochrome *bc*_1_ and interaction with nearby magnetic nuclei of protein surroundings (tables 1 and 2). We reflect on new mechanistic perspectives offered by these discoveries.

**Table 1.** Comparison of spectroscopic features of different semiquinones reported for the Q_o_ site. n.s., data not shown in the paper or experiment not performed; n.a., not applicable; SQ_o_, semiquinone in the Q_o_ site; SQ_i_, semiquinone in the Q_i_ site; SQ_res_, stigmatellin-insensitive radical signal.

properties of the signal/signals		SQ_o_ reported by					
		de Vries *et al*. [31]	Zhang *et al*. [33]	Cape *et al*. [34]	Vennam *et al*. [35]	Sarewicz *et al*. [36]	
						SQ_o_ uncoupled	SQ_o_–FeS spin-coupled
transience of signal		n.s.	yes	n.s.	n.s.	yes	yes
sensitivity to specific Q_o_-site inhibitors:	stigmatellin	n.s.	yes	yes	yes	yes	yes
	myxothiazol	n.s.	no	n.s.	n.s.	yes	yes
	strobilurins	n.s.	n.s.	n.s.	n.s.	yes	yes
presence of stigmatellin-insensitive residual signal		n.a.	n.a.	yes	yes	no	no
*g*-factor of central line		2.005	2.0040	2.0054	2.0044^a^	2.005	1.94
linewidth of X-band spectrum of SQ_o_ [G]		8.3	11.7	11.9	11.6	14.2	n.a.
microwave power saturation		saturable; slower relaxation than SQ_i_	saturable at 130 K; similar to SQ_i_	saturable at 77 K; similar to SQ_res_	n.s.	non-saturable at 200 K	n.s.
temperature-dependence of the EPR spectrum amplitude		n.s.	n.s.	n.s.	n.s.	anti-Curie behaviour	n.s.
spin–spin exchange interactions		no	no	no	no	no	yes
dipole–dipole interactions with:	reduced FeS	no	no	no	no	possibly yes	n.a.
	oxidized haem *b*_L_, *c*_1_	no	no	no	yes	yes	yes^b^
interaction with magnetic nuclei	hydrogen	n.s.	n.s.	deprotonated	deprotonated	n.s.	n.s.
	nitrogen	n.s.	n.s.	no	no	n.s.	n.s.

^a^Reported for yeast cytochrome *bc*_1_.

^b^Interaction between SQ_o_–FeS coupled centre and oxidized haem *b*_L_ is inferred from pulse EPR measurements [37].

**Table 2.** Comparison of the experimental conditions of trapping and measurements of semiquinone in the Q_o_ site. n.s., not shown or not performed; SMP, submitochondrial particles; cyt., cytochrome; *R. caps., Rhodobacter capsulatus*; *S. cerev., Saccharomyces cerevisiae.*

		de Vries *et al*. [31]	Zhang *et al*. [33]	Cape *et al*. [34]	Vennam *et al*. [35]	Sarewicz *et al*. [36]	
						SQ_o_ uncoupled	SQ_o_–FeS spin-coupled
aerobic (A) or anaerobic (AN)		A	AN	AN	AN	A	A
external oxidant		cyt. *c*	cyt. *c*_2_	none	cyt. *c*	cyt. *c*	cyt. *c*
isolated protein (I) or membranes (M)		M (SMP)	M	I	I	I	I
source/organism		beef heart	*R. caps.*	*R. caps.*	*R. caps.* and *S. cerev.*	*R. caps.*	*R. caps.*
temperature of detection (K)	CW EPR	50	130	77	77	105–210	20
	pulsed EPR	n.s.	n.s.	60	10–100	n.s.	10, 20
Q_i_ site blocked by antimycin		yes	yes	yes	yes	yes	yes

## 2. First report of antimycin-insensitive semiquinone signal on submitochondrial particles

In 1981, de Vries *et al.* [31] reported the detection of a new SQ in antimycin-inhibited submitochondrial particles under conditions of oxidant-induced reduction of haems *b* initiated by addition of fumarate/succinate to the membranes. This SQ signal was antimycin-insensitive but disappeared after addition of British anti-Lewisite—a thiol-containing compound that disrupts the Rieske cluster in cytochrome *bc*_1_ and abolishes activity of the Q_o_ site. Spectral properties of this SQ were different from the antimycin-sensitive SQ signal originating from the Q_i_ site (SQ_i_). This new SQ had clearly slower spin-lattice relaxation rate than SQ_i_ and exhibited smaller linewidth; the reported values were 8.3 and 10 G for the new SQ and SQ_i_, respectively. It should be noted that subsequent literature reported the linewidth of approximately 8.5 G for SQ_i_ signal [24,38,39].

The possible sensitivity of the antimycin-insensitive SQ to specific inhibitors of the Q_o_ site was not tested by the authors of the original report. However, the later work by Rich and co-workers [32] showed that under similar experimental conditions this SQ signal was not sensitive to inhibitors that block the activity of the Q_o_ site (myxothiazol, MOA-stilbene or stigmatellin), but at the same time, it was at least partially sensitive to several inhibitors of complex I and II.

## 3. Light-induced transient semiquinone in photosynthetic membranes

**Figure 2.**
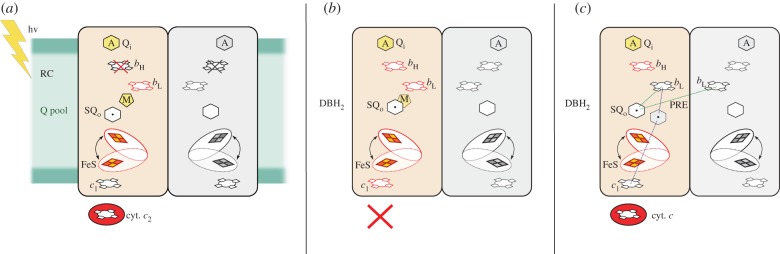
Schematic of the SQ intermediate trapped in the Q_o_ site with the corresponding enzyme state as reported in (*a*) [33], (*b*) [34] and (*c*) [35]. The redox states of cytochrome *bc*_1_ cofactors (FeS and haems) were either reduced or oxidized (red or black contour, respectively). In all cases, the Q_i_ site was occupied by antimycin (A). Myxothiazol (M) did not preclude the SQ_o_ trapping in (*a*). In (*b*), the authors speculated that SQ_o_ is formed in the vicinity of myxothiazol binding site. In (*c*), dotted green lines denote the possible dipole–dipole interaction of SQ_o_ with haems what lead to paramagnetic relaxation enhancement (PRE) of SQ_o_. The analysis of PRE resulted in assigning two possible locations of for SQ_o_ within the Q_o_ site. For simplicity, the second cytochrome *bc*_1_ monomer was shaded. In (*a*) the reaction was initiated by light activation of reaction centre (RC), while in (*b*) and (*c*) the reaction was initiated by injection of the reduced ubiquinone analogue (DBH_2_).

In 2007, Dutton and co-workers [33] generated SQ_o_ in chromatophore membranes of photosynthetic bacterium *Rhodobacter* (*R.*) *capsulatus*, which consisted of a complete cyclic electron transfer system that can be activated by light. In this system, cytochrome *bc*_1_ is coupled to photosynthetic reaction centre via cytochrome *c*_2_ and ubiquinone pool (figure 2*a*). The authors predicted that SQ_o_ should be visible at high pH which lowers the redox-midpoint values of the quinone couples provided that multiple flashes are delivered to mostly oxidized *c*-chain. The key to promoting SQ_o_ was to use the haem *b*_H_ knockout in which the *b*-chain can accept only one electron [14]. Indeed, with the help of these predictions, they detected flash-induced SQ in this mutant which, based on its properties, was assigned as SQ_o_. The radical signal at *g* = 2.004 was detected by EPR after freezing of the light-induced samples, and the amplitude of the signal was different depending on the time delay before freezing suggestive of its transient character. The signal was sensitive to stigmatellin, a potent inhibitor of the Q_o_ site, but not to myxothiazol—another inhibitor of the Q_o_ site. To explain the differential sensitivity to the two inhibitors, the authors assumed that in the case of myxothiazol, the inhibitor and ubiquinone bind simultaneously. In this mode, the residual activity of the Q_o_ site (interaction of ubiquinone with Rieske cluster) can still generate SQ_o_. The idea of a simultaneous presence of ubiquinone and myxothiazol within the Q_o_ site is inspired from crystallographic data which show that inhibitors can bind to distinctly different domains of the Q_o_ site: stigmatellin forms hydrogen bond with histidine ligand of FeS cluster while myxothiazol binds closer to haem *b*_L_ [40]. Furthermore, simultaneous binding of ubiquinol and β-methoxyacrylate inhibitors or binding of two molecules of ubiquinol was implicated from biochemical work [41,42] and more recent NMR studies [43]. However, recent data obtained from molecular dynamics (MD) simulations of cytochrome *bc*_1_ suggest that the Q_o_ site is a rather compact cavity and binding of additional quinone-like molecule next to the ubiquinol is energetically unfavourable [44].

To ascertain that the stigmatellin-sensitive signal originated form the Q_o_ site but not from other ubiquinone reactive protein, the authors tested conditions where oxidizing power of high potential c-chain was severely limited by slowing the electron transfer through haem *c*_1_ by orders of magnitude. As predicted, the light-induced SQ was not observed under those conditions, confirming that efficient outflow of electrons from Q_o_ through the c-chain is necessary for SQ_o_ generation.

The SQ_o_ spectrum, having an EPR linewidth of 11.7 G, appeared broader than the spectrum of SQ formed at the Q_i_ site (8.5 G). To explain the greater width of SQ_o_ spectrum, the authors considered the possibility of magnetic interactions with reduced Rieske cluster. This should manifest itself in a difficulty to saturate the CW EPR signal of SQ_o_which, however, was not observed experimentally. Factors other than interaction with fast-relaxing paramagnetic centre that would explain the greater linewidth of the SQ_o_ signal include greater g-tensor anisotropy [39] and/or hyperfine interactions with nearby magnetic nuclei [45] that are not resolved in CW EPR spectra at X-band.

## 4. Destabilized semiquinones in the Q_o_ site detected in isolated cytochrome *bc*_1_

Two publications by Kramer and co-workers [34,35] reported detection of SQ in the Q_o_ site in isolated antimycin-inhibited bacterial and yeast cytochrome *bc*_1_ under anaerobic conditions. In 2007, SQ was observed in the samples of *R. capsulatus* cytochrome *bc*_1_ freeze-quenched 10 ms after mixing with ubiquinol analogue—decylubiquinol (DBH_2_). Because cytochrome *c* was absent (figure 2*b*) [34], to initiate the reaction at the Q_o_ site, a significant fraction of Rieske cluster and cytochrome *c*_1_ must have been in the oxidized state prior to mixing. This, however, is problematic given the relatively high redox midpoint potentials of these two cofactors and the fact that the experiments were carried out under anaerobic conditions. Native cytochrome *bc*_1_ in this species, without any external oxidant added, typically shows 70–80% reduction level of cytochrome *c*_1_ while significantly lower reduction levels may indicate some structural distortions or protein damage.

While the EPR radical signal was generally sensitive to stigmatellin, approximately 30% of the signal (SQ_res_) still remained in the presence of this inhibitor. SQ_res_ shared some of the characteristics of stigmatellin-sensitive signal which was assigned as SQ_o_. Both SQ_o_ and SQ_res_ signals were broader than the signal of SQ_i_ and both showed similar power-saturation profiles. On the other hand, addition of exogenous relaxation enhancer (Ni^2+^ ions) suggested that the SQ_res_ was more exposed to the aqueous phase. For that reason, SQ_res_ was assigned to non-enzymatic oxidation of DBH_2_ in solution. However, as the experiment was performed in the absence of oxygen, this oxidation could not have been associated with O_2_. Rather, one can envisage that SQ_res_ formation might have been a result of a comproportionation. SQ_res_ exhibited different proton electron nuclear double resonance (ENDOR) spectrum from the SQ chemically induced in buffer (SQ_chem_). At the same time, SQ_o_ signal was reported to have indistinguishable CW EPR spectrum from the chemically produced SQ_chem_. Both SQ_o_ and SQ_res_ showed decreased amplitudes (greater than 10-fold) in the presence of molecular oxygen. The signals were not seen in the *bc*_1_ subcomplex (a complex of cytochromes *b* and *c*_1_ but lacking FeS subunit [46]).

Analysis of proton ENDOR spectra indicated that all three types of SQs (i.e. SQ_o_, SQ_res_ and SQ_chem_) were in the anionic form. This was inferred from the observation that hyperfine coupling constant of five-methyl group to the SQ electron spin in all three cases was different from the values characteristic for protonated/neutral SQs. A contribution of central line in SQ_o_ and SQ_res_ ENDOR spectra was different from that found in the spectrum of SQ_chem_ which was taken as indication that both SQ_o_ and SQ_res_ are located in the environment of lower proton concentration comparing with the aqueous phase of the SQ_chem_ environment. Electron spin echo envelope modulation (ESEEM) spectra showed no indications that SQs form hydrogen bonds with amide group of polypeptide chain nor histidine residues. Importantly, the properties of SQ_o_, including power saturation behaviour, did not reveal signs of dipolar magnetic interactions between SQ_o_ and neighbouring paramagnetic cofactors of the Q_o_ site, such as reduced FeS or oxidized haem *b*_L_. This, together with the confusing, in our view, properties of SQ_o_ versus SQ_res_ and problematic initial state of the enzyme raise concern about the origin of the signals.

In 2013, Kramer and co-workers [35] described SQ_o_ trapped using a method similar to that described previously [34], except that this time cytochrome *c* was added to provide oxidizing power to the *c*-chain and initiate the reactions in the Q_o_ site (figure 2*c*). While the width of new EPR signal of SQ_o_ was similar to that reported previously, the relaxation properties were clearly different. The spin echo of SQ_o_ decayed (2p-ESEEM experiment) much faster in comparison with SQ_chem_ signal in buffer which indicated that this time, unlike the previous case, the SQ_o_ interacted with fast-relaxing paramagnetic species. The authors concluded that the paramagnetic species that affect SQ_o_ are haems nearest to SQ_o_ which, based on simulations, were proposed to be either two haems *b*_L_ (each coming from individual monomers of cytochrome *bc*_1_ dimer) or haem *b*_L_ and haem *c*_1_ (both coming from the same monomer; figure 2*c*). However, no spectroscopic data verifying the oxidation state of haems were provided, nor relaxation rates for haems used in simulations, which are crucial parameters in determining distances by the use of relaxation enhancement [47,48]. The FeS cluster was excluded because of its slow relaxation when compared with haems at the temperature used in the experiments.

While the new SQ signal was generally sensitive to stigmatellin, around 30% of the signal was still observed in CW EPR spectra in the presence of substoichiometric concentration of this inhibitor. The sensitivity to other Q_o_ site inhibitors was not reported and it was not shown whether this new SQ signal disappears in the control mutants with inactive Q_o_ site. The overall shape of SQ_o_ proton ENDOR spectrum was similar to those reported previously for SQ_o_ and SQ_res_ indicating that SQ_o_ was deprotonated. Nevertheless, the splitting of doublet signals flanking the distant protons peak in ENDOR spectra was clearly larger than previously reported [34] implying that the detected SQs were in different environments.

The analysis of 4p-ESEEM spectra combined with the lack of the signal of nitrogen in 2p-ESEEM indicated that SQ_o_ was not hydrogen-bonded to the protein. Comparison of bacterial and yeast cytochrome *bc*_1_ did not reveal any spectral differences which indicated that SQ_o_ in both cases is the same chemical species trapped in similar environment.

The properties of SQ_o_ that emerged from ESEEM and ENDOR data led the authors to propose a model of ‘electrostatic cage’ trapping deprotonated SQ_o_. In this model, SQ is destabilized by lack of specific binding through hydrogen bonds or salt bridges. Insulating dielectric cage blocks the proton uptake back to SQ_o_ which secures that it does not leave the site. At the same time, the cage is supposed to prevent escape of any superoxide anion (or SQ) formed in the site. However, the destabilized SQ_o_ is proposed to conserve sufficient redox energy to reduce haem *b*_L_ which seems difficult to reconcile with the statement that SQ_o_ interacts paramagnetically with the oxidized haem *b*_L_. Furthermore, it is important to bear in mind that in photosynthetic reaction centres a similar concept of low dielectric gate around the SQ binding site was introduced to rationalize high stability of SQ, because the contributions from electrostatic energy and hydrogen bonds were not enough to explain SQ stabilization [49].

## 5. Semiquinone uncoupled and spin–spin coupled to Rieske cluster in isolated cytochrome *bc*_1_

**Figure 3.**
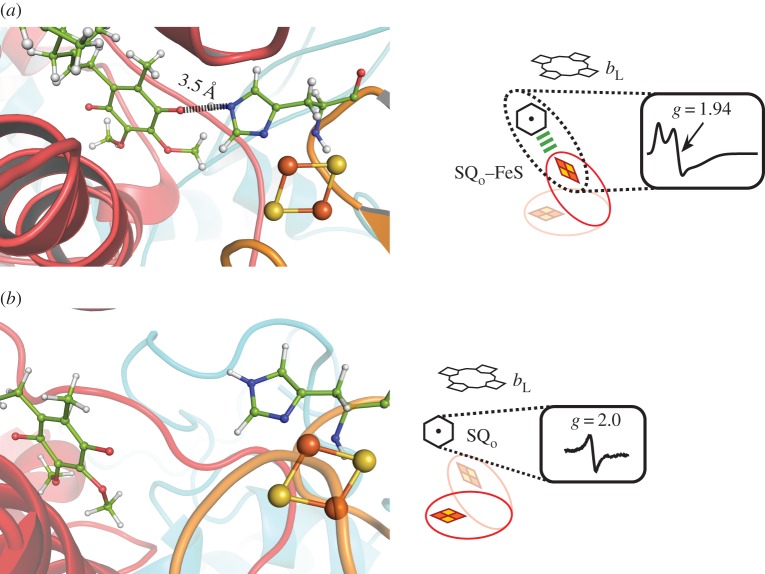
Structural model explaining the existence of two populations of SQ_o_ detected by CW EPR [36]. (*a*) Reduced FeS cluster is in the Q_o_ site and putative hydrogen bond between SQ_o_ and the cluster liganding histidine 156 (*R. capsulatus* numbering) facilitates spin–spin exchange interaction. (*b*) Increasing the distance between SQ_o_ and the FeS cluster owing to the dynamics of the FeS head domain and/or semiquinone within the site abolish the spin–spin exchange interaction and only dipole–dipole magnetic interactions between SQ_o_ and neighbouring fast-relaxing metal centres are possible. Structures shown in left panel are based on MD simulations [44].

In 2013, our group reported a discovery of two EPR transitions associated with the activity of the Q_o_ site [36]. Those transitions revealed the presence of two distinct populations of SQ_o_ formed at this site. The first signal at *g* = 1.94 was assigned as one of the transitions originating from the spin–spin exchange of two unpaired electron spins: one coming from SQ_o_ and the other from the reduced Rieske cluster (figure 3*a*). The second transition near *g* = 2.0 corresponded to the population of SQ_o_ for which the spin–spin exchange did not exist or was too weak to be resolved (figure 3*b*). Both populations were observed in samples of isolated, antimycin-inhibited cytochrome *bc*_1_ of *R. capsulatus* exposed to substrates, DBH_2_ and oxidized cytochrome *c*, under aerobic conditions. The changes in the amplitudes for these two signals (radical at *g* = 2.0 and SQ_o_–FeS spin-coupled centre at *g* = 1.94) during the catalytic turnover can be divided into two time regions. In the first (earlier) region, the amplitudes increase until they reach maximum, whereas in the second (later) region, the amplitudes progressively decrease to zero at the time point when the system reaches equilibrium.

Both signals were sensitive to stigmatellin and several other Q_o_-site-specific inhibitors (including myxothiazol and various synthetic strobilurins). Both signals were not observed in specific mutants that disabled activity of the Q_o_ site (such as cyt*b*:G158 W) [42,50] or the *bc-*subcomplex [46]. Moreover, in the presence of these inhibitors or mutations, no residual radical signals were detected. On the other hand, in +2Ala mutant (a mutation that makes the FeS head domain stay at Q_o_ site for prolonged time), the signal amplitude was higher compared with the native protein. More recent experiments indicate that both signals can also be generated under anaerobic conditions and that the characteristic *g* = 1.94 can be observed in native chromatophore membranes of *R. capsulatus* [37].

We proposed that the two populations of SQ_o_ reflect two configurations of the Q_o_ site. The spin–spin exchange (*g* = 1.94) by its nature has a clear distance constraint and can take place only when SQ_o_ and Rieske cluster are in proximity, as shown in figure 3*a*. In this configuration, a formation of a hydrogen bond between histidine residue coordinating Rieske cluster and ubiquinone molecule is possible. At larger distances (figure 3*b*) or upon breaking the putative hydrogen bond between SQ_o_ and histidine ligand, spin–spin exchange disappears and SQ_o_ becomes detected as a separate free radical species having a signal near *g* = 2.0. Nevertheless, in this case, SQ_o_ exhibited unusually fast relaxation compared with the relaxation of chemically generated SQ in buffer (by auto-oxidation of DBH_2_ in alkaline pH), which was expected given that the SQ_o_ is located in proximity to fast-relaxing paramagnetic metal centres of the Q_o_ site: oxidized haem *b*_L_ [51] and/or reduced FeS [52]. The fast spin-lattice relaxation of SQ_o_ manifested itself in significant homogeneous broadening of the EPR lines (both at X and Q band), the inability to saturate it with microwave power, and the presence of a Leigh effect (decrease in amplitude without apparent line broadening upon decrease of temperature). All these specific properties differentiated this SQ_o_ from the radical signals described in [33–35].

The two populations of SQ_o_ were incorporated to the model of electronic bifurcation of the Q_o_ site. We envisaged that the SQ_o_–FeS (*g* = 1.94) form might represent an initial step of ubiquinol oxidation when oxidized FeS withdraws an electron from ubiquinol. This state evolves into the state where SQ_o_ and reduced FeS exist as separate identities (distinguished by separate spectra, one of which is radical *g* = 2.0) before reduction of haem *b*_L_ by SQ_o_ takes place to complete the oxidation of QH_2_ at this site.

## 6. Semiquinone intermediates in relation to proton management of the Q_o_ site

The process of uptake and release of protons is an inherent part of redox chemistry of ubiquinones. As the energy of the SQH_2_^+^ (double protonated SQ) is very high [53], at least one proton needs to be released during oxidation of QH_2_ to make transfer of the first electron possible. Accordingly, in the ubiquinol oxidation at the Q_o_ site, a release of one or two protons is often considered to be a step initiating the entire reaction [15,54,55]. While the proton paths are largely unknown for the Q_o_ site, the detected SQ_o_ intermediates offer interesting new insights into this issue.

**Figure 4.**
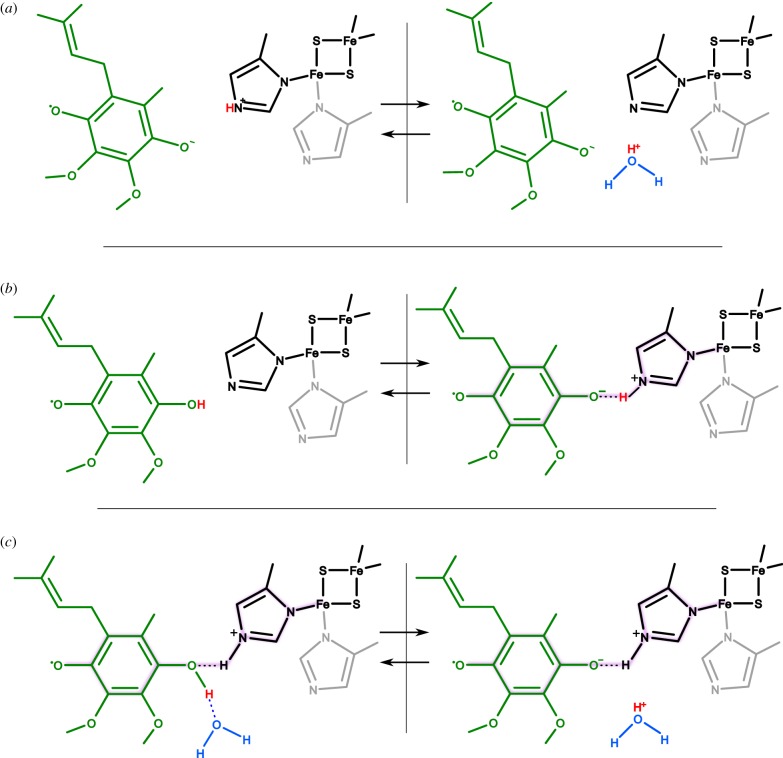
Different possibilities of proton involvement in the interactions of SQ_o_ (green) with the cluster liganding histidine (black) and/or water (blue). (*a*) SQ_o_ anion does not form a hydrogen bond with the histidine that reversibly exchange proton (red) with water molecules (arrows represent the reversibility of the reaction). (*b*) Protonated SQ_o_ reversibly donates proton to histidine which results in formation of hydrogen bond between this histidine and SQ_o_ anion. (*c*) Neutral SQ_o_ forms hydrogen bond with protonated histidine, whereas the proton originating from SQ_o_ is exchanged with water molecule. All three cases (*a–c*) may exist in an equilibrium but only in cases (*b*, right-hand panel) and (*c*, both panels) is a relatively strong spin–spin exchange interaction expected between SQ_o_ and the FeS cluster (magenta shadow shows the possible paths for the electron spin exchange).

The radicals with typical *g* = 2.0 are believed to be in a deprotonated/anionic form. Thus, it is plausible to expect that these SQs are relevant to a state having the two protons already released (here we consider the direction of ubiquinol oxidation; figure 4*a*). However, the spin–spin exchange state (*g* = 1.94), which most likely involves the hydrogen bond between histidine ligand of Rieske cluster and ubiquinone molecule, could represent a state before the proton release. For this state, at least two scenarios are possible.

The first scenario would accommodate an early model of proton pathway which proposed that initially deprotonated histidine ligand of Rieske undergoes protonation upon formation of hydrogen bond with ubiquinone molecule to subsequently withdraw the first proton from ubiquinone [55,56]. This hydrogen bond could be a good candidate for an element of the spin–spin exchange configuration (*g* = 1.94; figure 4*b*). This model, however, requires that histidine residue is maintained by the enzyme in a deprotonated form before it reacts with ubiquinol, which, as discussed in [44,57], is disputable.

The attractive alternative emerges from recent MD simulations which indicate that water molecules in the Q_o_ site can directly accept protons from ubiquinol upon its oxidation [44]. In this scenario, water molecules form hydrogen bonds with ubiquinol molecule. While protonated waters are short-lived, they may form an easy path for protons out of the protein through the cavity filled with water molecules. Hydrogen bond is also formed between histidine residue (protonated) and ubiquinol molecule but this bond is not involved in proton transfers from ubiquinol to the aqueous phase. This hydrogen bond may also serve as an inherent part of the configuration supporting spin–spin exchange between SQ_o_ and FeS cluster (figure 4*c*). Unlike the first scenario, this model allows the hydrogen-bonded configuration for spin–spin exchange to be assembled independently of ubiquinol proton stripping events.

## 7. Emerging questions about stability of SQ_o_ and its reactivity with oxygen

Quinones in solution under equilibrium undergo comproportionation (reverse of disproportionation) reaction according to the scheme [23,58]:
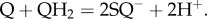
The equilibrium constant *K*_s_ for this reaction is often referred to as the stability constant for SQ which depends on the redox potentials of Q/SQ and SQ/QH_2_ pairs (E_Q/SQ_ and E_SQ/QH_2__, respectively):
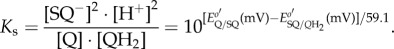
For ubiquinone-10, the redox potentials of *E*_Q/SQ_ and *E*_SQ/QH_2__ couples at pH 7 are −230 and +190 mV in bulk solution [23], respectively, which means that *K*_s_ is around 10^−8^. When concentrations of both Q and QH_2_ are, for example 100 μM, then equilibrium concentration of SQ^−^ is approximately 30 nM, which is at the lower limit of concentration needed to detect this species by EPR spectroscopy.

The *K*_s_ has a strict sense when considering comproportionation/disproportionation of Q/SQ/QH_2_ triad under equilibrium in solution but it is often used to describe the stability of SQ that can be formed within the Q_o_ sites of cytochrome *bc*_1_ even though species formed in the catalytic sites are insulated from bulk solution and they are unable to disproportionate directly [59]. Since original Mitchell's description of the Q cycle, SQ_o_ has been considered as highly unstable with the low stability constant *K*_s_ of the order of 10^−7^ or less [13,30,32,42,59,60]. This however remains an open question in the light of the SQ_o_ detections which report signals in the range from 1% up to 17% of the total Q_o_ sites. Cape *et al*. reported that SQ_o_ occupies 0.01–0.1 Q_o_ sites per monomer [34]. Given the total concentration of 10 μM for both cytochrome *bc*_1_ and QH_2_, it is possible to calculate the value of *K*_s_ around 10^−2^. Similar calculations performed by Sarewicz *et al*. give the estimated *K*_s_ of the order of 10^−2.6^ [36]. These values are in agreement with measured concentration of radicals reported for chemically modified SQs in solutions (10 µM of chloride-substituted quinone produces 260 nM of SQ with *K*_s_ that is larger than 10^−2^) [61]. Such relatively large values of *K*_s_ suggest some kind of stabilization of SQ_o_ in comparison with bulk solutions. We emphasize, however, a potential difficulty in describing stability of SQ_o_ using the *K*_s_ parameter, because all reported SQ_o_ signals in cytochrome *bc*_1_ were detected under non-equilibrium conditions for which the *K*_s_ parameter defining thermodynamic equilibrium may not be valid.

Considering the properties and conditions of trapping (table 1 and 2, respectively) the SQ_o_ intermediates summarized above it appears as if different SQ species for the Q_o_ site have been reported. A feature that unites all these reports is the observation that SQ_o_ cannot be detected in cytochrome *bc*_1_ unless the Q_i_ site is inhibited by antimycin or haem *b*_H_ is knocked-out by mutation. This effectively impedes re-oxidation of haem *b*_L_ through the path involving haem *b*_H_/Q_i_. It thus appears that the state with reduced haem *b*_L_ is required as condition for increasing probability of trapping SQ_o_. In reversibly operating Q_o_ site, SQ_o_ can, in principle, be formed in two ways and both indeed require reduced haem *b*_L_ as an initiation [62–66]. In a semiforward reaction, reduced haem *b*_L_ prevents electron transfer from SQ_o_ to haem *b*_L_ after oxidized FeS initially withdraws one electron from QH_2_ forming SQ_o_. In a semireverse reaction, reduced haem *b*_L_ initiates SQ_o_ formation by electron transfer to Q. In this context, the properties of SQ_o_ in [33,34] suggest that SQ_o_ was formed along with reduced haem *b*_L_ pointing towards the semiforward reaction scheme. On the other hand, the properties of SQ_o_ in [36] and [35] indicate that it was trapped along with oxidized haem *b*_L_ which points towards the semireverse reaction scheme. This mechanism is also supported by the observation that the rate of superoxide generation has a bell-shaped dependence on Q/QH_2_ ratio [67,68].

Interestingly, the semireverse reaction has recently been considered as the one leading to formation of SQ_o_ that can interact with oxygen and thus is responsible for generation of superoxide by the Q_o_ site [63–65]. In this scheme, unlike in a semiforward scheme, SQ_o_ can be formed in the configuration of the Q_o_ site that misses the second cofactor necessary to complete the reaction. The missing cofactor is the FeS cluster embedded in the head domain which during the catalytic cycle naturally undergoes movement between the Q_o_ site and haem *c*_1_ (outermost cofactor of the c-chain) [69]. It is thus possible that Q is reduced by haem *b*_L_ at the time when FeS cluster occupies positions remote from the Q_o_ site and is unable to immediately engage in electron transfer reaction with SQ_o_. This increases the probability of reaction of SQ_o_ with oxygen (if all electron transfers compete kinetically), as indeed implicated experimentally [64,65,68].

The presumed high reactivity of SQ_o_ with oxygen implies that anaerobic conditions should promote trapping SQ_o_. The reports of detection of SQ_o_ signals under anaerobic condition follow this expectation [33–35]. In one of these cases, it was additionally recognized that SQ_o_ could not have been observed under aerobic conditions [34]. There was also another report of failure to detect SQ_o_ in the presence of molecular oxygen, but those experiments were performed using freeze-quenched samples of cytochrome *bc*_1_ non-inhibited by antimycin [20]. However, the report of detection of two populations of SQ (*g* = 1.94 and *g* = 2.0) concerned aerobic conditions [36]. The relatively large quantities of SQ_o_ (spin–spin coupled to the Rieske cluster) detected under these conditions suggest that SQ_o_ is not as highly reactive with oxygen as commonly presumed at least in the presence of the spin exchange. In addition, high levels of SQ_o_ were observed in +2Ala mutant, which does not produce any detectable superoxide [64], implying that conditions of spin–spin coupling between SQ_o_ and FeS (*g* = 1.94) might be protective against superoxide generation.

## 8. Concluding remark

The assumption about extremely low *K*_s_ of SQ_o_ has traditionally been used to explain the long-standing difficulty in experimental detection of SQ_o_. We now seem to face the opposite situation where several seemingly different SQ_o_ intermediates have been exposed. The differences concern both the properties of SQ_o_ species and the experimental conditions used to trap the SQ_o_ intermediates. In our view, this certainly does not make it easier for a general reader to follow the progress in understanding the mechanism of ubiquinol oxidation at the Q_o_ site as it leaves space for various interpretations and mechanistic considerations that at this stage do not seem to converge into one generally accepted model of action. It remains to be seen whether the detected SQ_o_ signals represent the same intermediate of the Q_o_ site, or rather reflect different states of the reaction scheme. Are all of them truly associated with the operation of the Q_o_ site? What is the role of haem *b*_L_ in the formation of SQ_o_ and superoxide production? How does the intermonomer electron transfer between the two haems *b*_L_ influence these reactions? The available set of data on SQs provides now a framework for further studies in which various hypotheses can be critically examined and verified. Hopefully, this will lead to the formulation of the integrated model of the Q_o_ site catalysis and its involvement in superoxide generation.

## Endnote

1 We use the ‘proton *translocation*’ term in the context of cytochrome *bc*_1_ catalysis to describe creation of proton gradient by coupled oxidation/reduction of ubiquinol/ubiquinone taking place at the opposite sides of the membrane which is a different mechanism from active ‘proton *pumping*’ via specific proton channels within protein interiors.
